# Effects of Single and Repeated Doses on Disposition and Kinetics of Doxycycline Hyclate in Goats

**DOI:** 10.3390/ani10061088

**Published:** 2020-06-24

**Authors:** Erdinc Turk, Orhan Corum, Ibrahim Ozan Tekeli, Fatih Sakin, Kamil Uney

**Affiliations:** 1 Department of Pharmacology and Toxicology, Faculty of Veterinary Medicine, University of Hatay Mustafa Kemal, Hatay 31060, Turkey; erdincturk48@gmail.com (E.T.); ozantekeli@hotmail.com (I.O.T.); fsakin@mku.edu.tr (F.S.); 2 Department of Pharmacology and Toxicology, Faculty of Veterinary Medicine, University of Kastamonu, Kastamonu 37200, Turkey; 3 Department of Pharmacology and Toxicology, Faculty of Veterinary Medicine, University of Selcuk, Konya 42031, Turkey; kuney@selcuk.edu.tr

**Keywords:** doxycycline, goat, oral, pharmacokinetics, repeated dose

## Abstract

**Simple Summary:**

Doxycycline is used to treat bacterial infections such as pneumonia, skin and soft tissue infections, urinary tract infections, salmonellosis, and colibacillosis in goats. In goats, the single intravenous and intramuscular pharmacokinetics of doxycycline are known. However, there is no information regarding oral pharmacokinetics. This study aims to determine the single and repeated pharmacokinetics, bioavailability, and accumulation of doxycycline hyclate. Although doxycycline hyclate exhibited low intramuscular and oral bioavailability, its oral administration with favorable properties such as weak accumulation, wide distribution volume and long elimination half-life can be useful in the treatment of infections caused by susceptible pathogens in goats.

**Abstract:**

The aims of this study in goats were to determine the pharmacokinetics of doxycycline hyclate following single intravenous (IV), intramuscular (IM) and oral administrations of 20 mg/kg and to evaluate the pharmacokinetics and accumulation of doxycycline hyclate after repeated oral administrations at a 20 mg/kg dose every 24 h for 5 days. Six healthy male goats were used for the study. The study was performed in four periods according to a longitudinal study with a 15-day washout period. Plasma concentrations of doxycycline were determined using HPLC-UV and analyzed by a non-compartmental method. IM injection of doxycycline caused swelling and pain due to irritation in the injection site. After IM and oral administrations, terminal elimination half-life (t_1/2λz_) and mean residence time (MRT) were prolonged and areas under the curve (AUCs) were low. The mean bioavailability of IM and oral administration was 51.51% and 31.39%, respectively. Following repeated oral administration, the accumulation ratio of doxycycline was 1.76. Pharmacokinetic properties including weak accumulation, wide distribution volume and long elimination half-life can make doxycycline hyclate valuable for repeated use via an oral route in the treatment of some infectious diseases in goats. However, the determination of pharmacodynamic effects on susceptible pathogens isolated from goats is also necessary to confirm the drug dosage regimen.

## 1. Introduction

Doxycycline (alpha-6-deoxytetracycline), a semi-synthetic derivative of oxytetracycline, has a broad spectrum of action including Gram-positive, Gram-negative, and atypical bacteria (*Mycoplasma* spp., *Chlamydophila* spp., *Legionella* spp., *Rickettsia* spp.) [1]. Doxycycline shows bacteriostatic activity by blocking the binding of aminoacyl-tRNA to the mRNA ribosome complex of bacteria, thereby inhibiting protein synthesis [2,3]. Compared to other tetracyclines, doxycycline has higher lipophilicity, longer elimination half-life, better tissue penetration, higher therapeutic efficacy, and a lower side effect profile [4]. In addition to its antibacterial effect, doxycycline has anti-inflammatory, antineoplastic, and matrix metalloproteinase inhibitory effects [1].

Doxycycline is used in treating respiratory tract infections, sexually transmitted infections, malaria prophylaxis, and rickettsial infections in humans [1,5]. The use of doxycycline for treating infections caused by susceptible bacteria in cattle, dogs, cats, pigs, poultry, and turkeys has been approved by the European Medicines Agency (EMA) [4]. This antibiotic is used to treat bacterial infections such as pneumonia, skin and soft tissue infections, urinary tract infections, salmonellosis, and colibacillosis in goats [6]. Doxycycline at a dose of 20 mg/kg is recommended for treating pneumonic pasteurellosis in goats [7].

In many animal species, the repeated use of doxycycline for 3–5 days is recommended for bacterial infections [8]. In goats, despite the established pharmacokinetics of doxycycline for different doses and formulations following single-dose intravenous (IV) and intramuscular (IM) administration, there is no information regarding its oral pharmacokinetics [9,10,11]. The aims of this study in goats were: (1) to determine the pharmacokinetics and bioavailability of doxycycline hyclate following single intravenous (IV), intramuscular (IM), and oral administrations of 20 mg/kg, and (2) to evaluate the pharmacokinetics and accumulation of doxycycline hyclate after oral administrations at a 20 mg/kg dose every 24 h for 5 days.

## 2. Materials and Methods

### 2.1. Chemicals

Doxycycline hyclate analytical standard was obtained from TCI (Tokyo Chemical Industry) in powder form. Acetonitrile high-pressure liquid chromatography grade was used, and all chemicals were obtained from Merck (Darmstadt, Germany). Doxycycline hyclate analytical standard was administered to goats after dissolving 100 mg/mL with injection water.

### 2.2. Animals

Six healthy male goats, weighing 30–40 kg and aged 1.6 to 2 y, were used. According to general clinical examination and the evaluation of their hematological and biochemical parameters, the goats were determined to be healthy. One week before the start of the study, goats were placed into pens where optimum conditions were met for acclimation and kept there during the entire study period. Goats were fed twice a day with antibiotic-free feed and were provided access to water and dry grass ad libitum. The experimental procedure on goats was approved (2018/10-3) by the Local Ethics Committee for Animal Research Studies at Hatay Mustafa Kemal University (Hatay/Turkey).

### 2.3. Experimental Procedure

The study was performed in four periods according to a longitudinal study with a 15-day washout period. Doxycycline hyclate at a dose of 20 mg/kg was administered to the six goats in each period of the study. Doxycycline hyclate in first, second and third periods was administered via IV (right jugular vein), IM (between semitendinosus and semimembranosus muscles), and oral (via an ororuminal tube followed flushing the tube with 0.5 L of water) routes, respectively, with the 15-day washout period. The IV administration was performed for 30–45 s to prevent the possible cardiotoxic effect of doxycycline [12]. In the IM administration, an equal volume of drug solution was injected into each of the left and right injection sites due to the high volume of drug solution. Blood samples of 1 mL were collected in heparinized tubes using a catheter placed into the left jugular vein before doxycycline administration (0 h) and at 5, 15, 30, and 45 min and 1, 2, 3, 4, 6, 8, 10, 12, 24, 36, and 48 h following administration.

In the fourth period of the study following the 15-day washout period, doxycycline hyclate was administered via the oral route every 24 h for 5 days (total five doses). Blood samples of 1 mL were collected into heparinized tubes from the jugular vein through a catheter at 0 (control), 5, 15, 30, and 45 min and 1, 2, 3, 4, 6, 8, 10, 12, and 24 h following the 1 (first) and 5 (last) doses; and by jugular venipuncture at 1, 8, and 24 h following drug administration on days 2, 3, and 4. After the blood samples were 4000× *g* for 10 min, the plasma obtained was stored at −80 °C until analysis.

### 2.4. Analytical Procedures

Plasma concentrations of doxycycline were determined using an HPLC-UV system by modifying the previously defined method [13]. Briefly, 250 µL of buffer/EDTA (0.1 mol/L disodium EDTA, containing 0.1 mol/L sodium phosphate) and 50 μL of perchloric acid (20%) were added to a test tube containing 200 µL of plasma. After vortexing for 2 min, the samples were 10.000× *g* for 10 min. An amount of 50 μL of supernatant filter was injected into an HPLC system after being filtered using a 0.45 µm syringe. The HPLC system consisted of an SPD-20A UV-Vis detector, an autosampler (SIL 20A), a pump (LC-20AT), a column oven (CTO-10A), and a degasser (DGU-20A). The flow rate was 1 mL/min. Doxycycline separation was performed with an ODS-3 column (5 µm, 250 × 4.6 mm I.D. column, GL SCI), maintained at 30 °C and the detection wavelength of 350 nm. The mobile phase consisted of acetonitrile (30%) and 0.01 mol/L trifluoroacetic acid in water (70%).

Doxycycline hyclate was dissolved in water to obtain a concentration of 1 mg/mL. The doxycycline calibration curves constructed using nine calibration standards in the range of 0.04–40 µg/mL were linear (R^2^ > 0.9995). Six replicates at the concentrations of 0.1, 1 and 10 μg/mL were used to determine recovery, precision, and accuracy. The recovery of doxycycline in plasma was ≥ 86%. The lowest limit of quantification (LLOQ) was 0.04 µg/mL, with <20% precision and ±15% accuracy. For both interday and intraday precision, the coefficient of variation was <5.3%. The bias for interday and intraday accuracy was ±5.7%.

### 2.5. Pharmacokinetic Calculations

Pharmacokinetic parameters for each goat were determined by non-compartmental analysis using the software program WinNonlin 6.1.0.173 (Pharsight Corporation, Scientific Consulting Inc., North Carolina, USA). The pharmacokinetic parameters calculated included area under the curve (AUC), terminal elimination half-life (t_1/2λz_), mean residence time (MRT), total clearance (Cl_T_), and volume of distribution at steady state (V_dss_). Mean absorption time (MAT) was calculated as MRT_IM,OR_−MRT_IV_. Doxycycline bioavailability (F) for IM and oral administrations was calculated using the following formula: F = (AUC_IM,OR_/AUC_IV_) × 100.

The peak plasma concentration (C_max_), minimum plasma concentration (C_min_) and time to reach C_max_ (T_max_) were assigned by direct observation of the plasma concentration–time curve of each animal. The accumulation ratio (R) of doxycycline in plasma following repeated oral administrations was calculated using R=AUC_(0–24)ss_/AUC_(0–24)1_, where AUC_(0–24)ss_ and AUC_(0–24)1_ were the areas under the curve calculated for steady-state at the last (day 5) and first (day 1) dose administration, respectively [14].

### 2.6. Statistical Analysis

Statistical analysis was performed using the SPSS 22.0 (IBM Corp, Armonk, NY, USA) program. All pharmacokinetic parameters are presented as mean ± SD. The difference in AUC after a single IV, IM, and oral administration was analyzed using one-way analysis of variance and post hoc Tukey’s test. The t_1/2λz_ and MRT are presented as harmonic mean ± SD, and the differences among routes of administration and between 1 and 5 doses following repeated oral administration were analyzed using Wilcoxon’s rank sum test. Differences in other pharmacokinetic parameters based on the route of administration and on doses 1 and 5 following repeated oral administration were evaluated using a paired *t*-test. A *p* value of <0.05 was considered statistically significant. 

## 3. Results

### 3.1. Safety

Adverse effects were not observed after single and repeated oral administration of doxycycline in goats. However, adverse effects such as tremor, tachypnea, excessive salivation, and prostration, which lasted for approximately 1 h, were observed following IV injection, and symptoms of pain such as shouting, lying down, restlessness, which lasted for approximately 6 h, and swelling at the injection site occurred following IM injection.

### 3.2. Single-Dose Study

**Figure 1:**
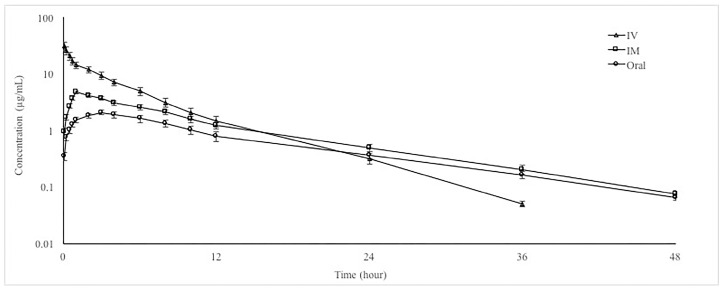
Semi-logarithmic plasma concentration–time curves of doxycycline hyclate following intravenous (IV), intramuscular (IM) and oral administrations at a dose of 20 mg/kg in goats (*n* = 6, mean ± SD).

Mean ± SD plasma concentration–time curves and the pharmacokinetic parameters of doxycycline after IV, IM, and oral administrations at a dose of 20 mg/kg in goats are presented in Figure 1 and Table 1, respectively. After IM and OR administrations, t_1/2λz_ and MRT were prolonged and AUCs were low.

### 3.3. Repeated Oral Dose Study

**Figure 2:**
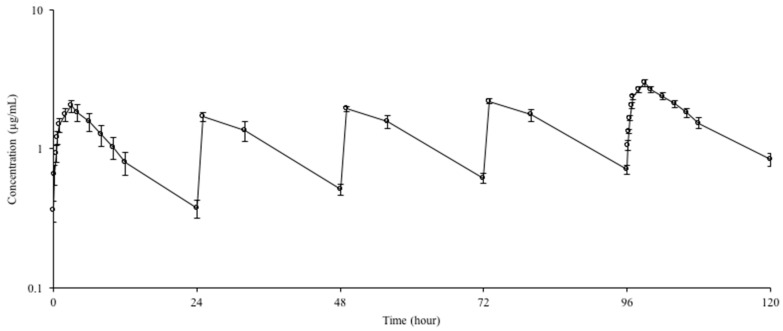
Semi-logarithmic plasma concentration–time curves of doxycycline hyclate following repeated oral administrations at the dose of 20 mg/kg every 24 h for 5 days in goats (*n* = 6, mean ± SD).

Mean ± SD plasma concentration–time curves and the pharmacokinetic parameters of doxycycline following repeated oral administrations at the dose of 20 mg/kg every 24 h for 5 days in goats are presented in Figure 2 and Table 2, respectively. In steady state (5 doses), t_1/2λz_ and MRT were prolonged and AUC, C_max_, and C_min_ increased. Following repeated oral administration, the R of doxycycline was 1.76 ± 0.24.

## 4. Discussion

In this study, the pharmacokinetics of doxycycline for single and repeated oral administrations were demonstrated for the first time in goats. At the present time, there is a commercial formulation of doxycycline hyclate for IM administration in goats [15]. We aimed to determine the pharmacokinetics of doxycycline after repeated IM and oral administrations in goats, but repeated IM administration was excluded due to the severe adverse effects of a single IM injection. It has been reported that IM injection of doxycycline causes swelling and pain due to irritation in goats, but no adverse effects have been observed in sheep [11,16]. In this study, single and repeated oral administrations of doxycycline in goats did not cause any adverse effects. Similar results have been reported in sheep [5]. The IV injection of doxycycline causes tachycardia, hypertension, collapse, and death in horses, whereas it causes sialism, tachypnea, tremors, and rear-end weakness in sheep [5,17]. The administration of doxycycline within 30–45 s is recommended to reduce the side effects occurring after IV administration [12]; however, goats in the present study showed adverse effects. Therefore, IV doxycycline can be administered as slower bolus over a longer period of time.

Doxycycline has a wide distribution volume (0.93–4.91 L/kg) in animals such as goats, sheep, calves, and dogs [5,18,19,20]. In this study, this antibiotic showed also wide distribution volume (1.15 ± 0.17 L/kg) in goats following IV administration. The binding ratio of doxycycline to plasma proteins in goats is 33%, whereas it is ≥90% in many animal species such as sheep, calves, dogs, and cats [5,10,18,19]. Doxycycline, which is more lipophilic than other tetracyclines, is present in much higher concentration in many tissues than in plasma and can accumulate in bone, tooth, and adipose tissues [1,4,10]. The wide distribution of doxycycline may be because of its lipophilic structure permitting good penetration into the tissues. The Cl_T_ and t_1/2λz_ of doxycycline after IV administration in goats were 0.22 ± 0.03 L/h/kg and 4.39 ± 0.14 h, respectively. In previous goat studies, the Cl_T_ and t_1/2λz_ of doxycycline were 0.41–0.71 L/h/kg and 4.11–4.62 h, respectively [10,11,21].

After IV, IM, and oral administration of doxycycline in goats, the t_1/2λz_ was 4.39 ± 0.14, 8.84 ± 0.52, and 9.81 ± 0.51 h, respectively, and the t_1/2λz_ was different among the routes of administration, with an order of OR > IM > IV. The t_1/2λz_ of doxycycline after IM administration is similar in goats and prolonged in calves compared to IV administration [11,20]. After extravascular administration in different drugs and animal species, prolonged t_1/2λz_ has been reported as compared to IV [22,23]. The MRT value obtained after IM and oral administrations is longer than that obtained after IV administration. The longer t_1/2λz_ of doxycycline after IM and oral administration than after IV administration may be related to the flip-flop phenomenon due to a longer MAT than MRT_IV_ [24]. In this study, the IM bioavailability of doxycycline was 51.51 ± 8.64%. However, in previous goat studies, IM bioavailability was ≥99% [10,11]. Following IM administration of doxycycline, edema and pain due to irritation have been reported as in our study [11]. The low IM bioavailability in the present study may be due to the varying degrees of irritation based on dose differences or different formulations used. In our study, doxycycline showed a low oral bioavailability of 31.39 ± 4.51%. Low oral bioavailability for doxycycline has been reported in sheep (36%) [5] and pigs (21%) [12]. If doxycycline is administered orally in humans, almost all of it is absorbed, and its oral bioavailability is ≥80%. Compared to other tetracyclines, food intake has less impact on the oral absorption of doxycycline and reduces plasma concentration by about 20% in humans [25]. In horses, foods have been reported to reduce the plasma concentration of doxycycline by about 50% (C_max_; from 0.97 to 0.43 µg/mL) and to increase T_max_ (from 0.75 to 4 h) [26]. In this study, there was no restriction on feed intake for the goats prior to oral drug administration. Therefore, the low oral bioavailability of doxycycline in goats might be due to feed intake.

In steady state (5 doses) following oral administration of doxycycline at a dose of 20 mg/kg, t_1/2λz_ and MRT were prolonged and AUC, C_max_, and C_min_ increased. Increased C_max_, AUC, and t_1/2λz_ have been reported when five doses of doxycycline were administered to horses every 12 h and to broilers every 24 h [13,26]. In repeated administrations, the plasma concentration of the drug increases until it reaches a steady state [27]. The value of R between 1.2 and 2 is defined as weak accumulation [28]. In this study, the repeated oral administration of doxycycline showed weak accumulation with R of 1.76. R after repeated administration of doxycycline has been reported as 1.11 in broilers and 1.96 (12-h interval) and 1.32 (24-h interval) in horses [13,26]. In this study, the C_max_ of doxycycline following five doses increased from 2.05 ± 0.22 to 2.96 ± 0.16 µg/mL. Doxycycline is well tolerated with an increased C_max_ from 1.60 to 5.56 µg/mL after IM administration at a dose of 5 to 20 mg/kg in goats [9,10,11]. The increased C_max_ of doxycycline within the therapeutic window after repeated oral administration may indicate a result with a good therapeutic efficacy.

*Escherichia coli*, *Pasteurella multocida*, *Streptococcus* spp., and *Staphylococcus aureus* pathogens cause diseases such as pneumonia, septicemia, and enteritis in goats [29]. The minimum inhibitory concentration (MIC) value of doxycycline has been established for *P. multocida* (MIC of 0.4 µg/mL) and *Mycoplasma agalactiae* (MIC_90_ of 0.8 µg/mL) isolated from goats; however, it has not been reported for other bacteria [7,30]. The MIC value of doxycycline for *E. coli* (ATCC 25922), *P. multocida*, *Streptococcus pneumonia* (ATCC 49619), and *S. aureus* (ATCC 25923) with test bacteria ranges within 0.09–1.5 µg/mL [31]. The treatment success of antibiotics based on their antibacterial activity depending on time or concentration is evaluated using the AUC/MIC, C_max_/MIC, and T>MIC pharmacokinetic/pharmacodynamic indices. The antibacterial activity of doxycycline depends on time when up to 2–4 times the MIC and concentration when up to 8–16 times the MIC [31]. The susceptible breakpoint and moderately resistant breakpoint values of doxycycline were 0.4 and 1.5 µg/mL, respectively [11]. The pharmacokinetic/pharmacodynamic indices in this study were calculated using these MIC values only for day five after the repeated oral administration of doxycycline. The AUC/MIC and C_max_/MIC of doxycycline were 100.75 and 7.40, respectively, for bacteria with the MIC value of 0.4 µg/mL, and 26.87 and 1.97, respectively, for bacteria with the MIC value of 1.5 µg/mL. For bacteria with the MIC value of 0.4 and 1.5 µg/mL, the T>MIC ratios of doxycycline administered at a dose of 20 mg/kg and a dose interval of 24 h were 152% and 54%, respectively. The AUC_0–24_/MIC ratios to achieve the bacteriostatic and bactericidal effect of doxycycline against *Haemophilus parasuis* in pigs have been determined as 59 and 98, respectively [32]. In the present study, an AUC_0–24_/MIC ratio of 100.75 for bacteria with the MIC value of 0.4 µg/mL was higher than that previously reported for optimum bacteriostatic and bactericidal activity [32]. In goats, a 20 mg/kg oral dose of doxyxycline can provide the efficient treatment of infections caused by susceptible bacteria with the MIC value of ≤0.4 µg/mL. However, a clinical investigation is necessary to determine the actual efficacy of dosage in goats with natural disease or disease models.

## 5. Conclusions

Doxycycline hyclate showed low bioavailability after IM and oral administration in goats. However, it exhibited favorable properties such as weak accumulation after oral administration, wide distribution volume and long elimination half-life. The single IM injection of doxycycline hyclate solution prepared in water caused severe local adverse effects in the injection site. The 24 h dosing intervals at a 20 mg/kg dose of doxycycline may provide an AUC/MIC of >100, C_max_/MIC of >7 and T>MIC of >100% in the treatment of infections caused by susceptible pathogens with the MIC value of ≤0.4 μg/mL in goats. However, the determination of the pharmacodynamic effects of doxycycline on susceptible pathogens isolated from goats is also necessary to confirm the drug dosage regimen.

## Figures and Tables

**Table 1:** Plasma pharmacokinetic parameters of doxycycline hyclate following intravenous (IV), intramuscular (IM) and oral (OR) administrations at a dose of 20 mg/kg in goats (*n* = 6, mean ± SD).

Parameter	IV	IM	OR
t_1/2λz_ (h) (HM)	4.39 ± 0.14 ^c^	8.84 ± 0.52 ^b^	9.81 ± 0.51 ^a^
AUC_0–24_ (h * µg/mL)	91.11 ± 13.90 ^a^	40.75 ± 4.02 ^b^	23.73 ± 3.22 ^c^
AUC_0–last_ (h * µg/mL)	92.83 ± 14.03 ^a^	46.22 ± 4.66 ^b^	28.01 ± 3.70 ^c^
AUC_0–∞_ (h * µg/mL)	93.15 ± 14.00 ^a^	47.18 ± 4.69 ^b^	28.92 ± 3.71 ^c^
MRT_0–∞_ (h) (HM)	5.26 ± 0.16 ^c^	11.71 ± 0.52 ^b^	13.97 ± 0.34 ^a^
MAT (h)	-	6.45	8.71
Cl_T_ (L/h/kg)	0.22 ± 0.03	-	-
V_dss_ (L/kg)	1.15 ± 0.17	-	-
T_max_ (h) (M)	-	1	3 *
C_max_ (µg/mL)	-	4.74 ± 0.28	2.09 ± 0.21 *
F%	-	51.51 ± 8.64	31.39 ± 4.51 *

^a,b,c^: Varied characters in the same row are statistically different (*p* < 0.05). * Significantly different from IM administration (*p* < 0.05). t_1/2λz_, terminal elimination half-life; AUC, area under the plasma concentration–time curve; MRT, mean residence time; MAT, mean absorption time; Cl_T_, total clearance; V_dss_, volume of distribution at steady state, T_max_, time to reach peak concentration; C_max_, peak concentration; F, bioavailability; HM, harmonic mean, M, median.

**Table 2:** Plasma pharmacokinetic parameters of doxycycline hyclate following repeated oral administrations at the dose of 20 mg/kg every 24 h for 5 days in goats (*n* = 6, mean ± SD).

Parameter	1-day	5-day
t_1/2λz_ (h) (HM)	9.73 ± 0.84	12.35 ± 0.66 *
AUC_0–24_ (h*µg/mL)	23.31 ± 3.33	40.30 ± 2.29 *
MRT_0–24_ (h) (HM)	14.24 ± 1.13	18.32 ± 1.24 *
T_max_ (h) (M)	3	3
C_max_ (µg/mL)	2.05 ± 0.22	2.96 ± 0.16 *
C_min_ (µg/mL)	0.38 ± 0.05	0.84 ± 0.09 *
R	-	1.76 ± 0.24

* Significantly different from 1 day (*p* < 0.05). t_1/2λz_, terminal elimination half-life; AUC, area under the plasma concentration-time curve; MRT, mean residence time; T_max_, time to reach peak concentration; C_max_, peak concentration; C_min_, minimum plasma concentration; R, accumulation ratio; HM, harmonic mean; M, median.
